# Dietary patterns and survival to 100 + years: an empty systematic review of cohort and case–control studies

**DOI:** 10.1186/s13690-022-00914-2

**Published:** 2022-06-29

**Authors:** Winnie Poulsen, Kaare Christensen, Christine Dalgård

**Affiliations:** 1 Epidemiology, Biostatistics and Biodemography, Department of Public Health, University of Southern Denmark, Odense C, Denmark; 2 Danish Aging Research Center, Epidemiology, Biostatistics and Biodemography, Department of Public Health, University of Southern Denmark, Odense C, Denmark; 3 Clinical Pharmacy, Pharmacology and Environmental Medicine, Department of Public Health, University of Southern Denmark, Odense C, Denmark

**Keywords:** Centenarians, Diet, Survival, Exceptional longevity, Ageing

## Abstract

**Background:**

Centenarians are used as a model of healthy ageing and longevity. Diet is a factor known to affect mortality in middle aged adults and elderly. However, it is unknown whether diet has an impact on survival to 100 + years. The aims of this systematic review were to summarize the evidence on (i) the association between dietary patterns in late adult life and survival to 100 + years and (ii) the common characteristics across dietary patterns that are shown to be positively associated with survival to 100 + years.

**Methods:**

We performed a systematic literature search in MEDLINE and EMBASE, and a hand search at four longevity projects homepages up to 4 June 2021. We searched for cohort and case–control studies investigating the association between dietary patterns and all-cause mortality among individuals aged ≥ 65 years at enrolment regardless of their health status and residence. Studies were excluded if follow-up was performed too soon to allow the population or a subgroup of it to have become 100 + years of age.

**Results:**

Of 3,685 identified records 108 reports were retrieved and full text screened. No studies met our inclusion criteria, thus the review process resulted in no eligible studies found. Hence, no risk of bias assessment and no synthesis of data was performed.

**Conclusions:**

No studies have investigated dietary patterns in late adult life in relation to survival to 100 + years of age. We have observed that as of June 2021 published cohort studies exist investigating all-cause mortality risk from different dietary patterns among the oldest old, but follow-up has been performed before the cohort could have reached 100 years of age. However, cohorts do exist where data on dietary habits in adult life has been collected decades ago and where follow-up in 2022 will allow the participants to have become 100 + years old.

**Registration:**

The review protocol is published at University of Southern Denmark’s Research Portal (Poulsen et al. Dietary Patterns and Survival to 100 + Years: Protocol for a Systematic Review of cohort and case–control studies University of Southern Denmark's Research Portal: University of Southern Denmark, 2021) available at https://portal.findresearcher.sdu.dk/en/publications/kostm%C3%B8nstre-og-overlevelse-til-100-%C3%A5r-protokol-for-en-systematisk. We have specified aim (i) of our research question in this report compared to the protocol, by adding “late” to “adult life”.

**Supplementary Information:**

The online version contains supplementary material available at 10.1186/s13690-022-00914-2.

## Background

Centenarians are being studied as a model for healthy ageing [1–4] aiding researchers in understanding the determinants of both exceptional health span and life span. Centenarians do not only exhibit intriguing exceptional survivorship. A large fraction of the population also seems to have lived independently into their nineties [5], and around 10–35% might continue to do so after reaching age 100 [4–6]. While a few centenarians might escape age-associated diseases, e.g., heart disease, diabetes, stroke, and various cancers [7], it has been suggested that the majority of centenarians do get affected by major diseases and either survive with or delay the onset of these [6, 7]. Understanding factors that has promoted this group of individuals’ longevity holds the potential for improving primary and secondary preventive efforts.

Human life span varies due to several factors. Studies of Danish twins estimate that genetic factors account for approximately 25% of the variation [8, 9]. This leaves 75% to be determined by a combination of random phenomena experienced throughout life and environmental factors, e.g., lifestyle factors [10]. Lifestyle factors including tobacco use, use of alcohol, physical activity, and diet are behaviours that are influencing life span in adult populations by affecting the risk of developing age-associated non-communicable diseases [11, 12]. Currently, these diseases are the main reasons for death at adult ages in high-income countries [13].

To assess the effects of overall dietary patters on morbidity and mortality outcomes, different approaches to measuring dietary patterns have been developed. One method is estimating adherence to predefined dietary guidelines operationalized as dietary quality indices (DQI). A plethora of DQI exists, e.g., the Mediterranean Diet Score, the Healthy Eating Index, and the Healthy Diet Indicator, and updated or adapted versions of these [14–16]. Through primarily epidemiologic research inverse associations with DQI and cardiovascular disease mortality, cancer mortality, as well as all-cause mortality have been documented [15, 16]. While some food components in DQI are common, e.g. vegetables and fruits, other components, such as olive oil, are not found in most of the DQI [15]. Dietary patterns have also been calculated by the means of factor or cluster analyses. These are *a posterior* data driven approaches, where dietary patterns are identified based on individuals sharing same dietary components, often resulting in two distinct patterns: prudent and Western [17]. Further, diet diversity scores (DDS) have been developed. Scores are derived by counting the number of different food groups or foods within groups consumed. The scores are intended to reflect how diverse the diet is and thus to which degree essential needs for nutrients are met [18]. Some DQI include this diversity or variety component, while other DQI indices reflect either intake of healthy or unhealthy dietary components, or both [15].

Embarking on the journey to elucidate the role of dietary patterns in centenarians’ health and exceptional longevity, surveys of centenarians’ diets have been conducted in different parts of the world, e.g. in Greece [19], China [20, 21], Italy [22], and Japan [23, 24]. However, attempting to establish if dietary behaviours can be regarded as one of the explanations to the exceptional survivorship of centenarians, it is essential to study adults’ dietary patterns years before they potentially become centenarians. This warrants longitudinal studies.

Findings from longitudinal studies investigating the association between adherence to healthy dietary patterns and survival among primarily European and American middle aged and younger elderly have been summarized in systematic reviews. Summarized healthy dietary patterns includes adherence to the Mediterranean Diet (MD) [25], the Dietary Approaches to Stop Hypertension, and diets scoring high on the Healthy Diet Index, and the Alternative Healthy Eating Index [16, 26, 27]. Meta-analyses of data on these dietary patterns have provided consistent results showing an association between adherence to these healthy diets and survival. Pooled estimates have showed that individuals within the categories of highest adherence to healthy dietary patterns have 20–22% lower all-cause mortality-risk than adults within the lowest healthy diet-scores groups [16, 25–27]. Further, it has been shown that one standard deviation increment in adherence to the MD is significantly associated with an 8% reduction in all-cause mortality risk [25]. Estimates were based on data from models in primary studies with relevant adjustment for a number of potential confounders, e.g., age, sex, smoking, biometrical measures, and prevalent or family history of diseases [16, 25–27].

It seems intuitive to extrapolate the protective effect of adhering to a healthy diet on mortality risk during adult life to old and exceptional old ages. However, it is suggested that the effect of some risk factors change in the highest ages [28–30]. Although far less studied, however, some evidence exists that the association between healthy dietary habits and survival in fact could persist into old age. One study among Chinese individuals aged ≥ 80 years documented a significant association between dietary habits and mortality risk. Diet quality was assessed using a diet diversity score (DDS), which was calculated by counting one DDS unit per 1 of 9 major food groups consumed “often or almost daily”. The authors report a trend of a 9% decrease in mortality risk by one unit increase in DDS after comprehensive adjustment [31].

We intend to evaluate the progress in this research field by elucidating if dietary behaviours are a determinant of achieving exceptional survivorship. To our knowledge no systematic review exists on dietary habits in relation to survival to 100 + years.

Therefore, this systematic review was done to summarize findings from published studies to investigate (i) the association between dietary patterns in late adult life and survival to 100 + years and (ii) the common characteristics across dietary patterns that are shown to be positively associated with survival to 100 + years.

## Methods

This systematic review was conducted in concordance with a pre-published study protocol [32]. It was reported according to the Preferred Reporting Items for Systematic Reviews and Meta-Analyses updated in 2020 (PRISMA 2020) [33, 34] where applicable as documented in the PRISMA 2020 Checklist (See Additional file 1). Additionally, we integrated advise from papers discussing how to report empty reviews [35–38].

### Search strategy

The search strategy was developed through four processes: (i) WP performed preliminary searches for systematic reviews on longevity, (ii) WP conducted crude searches for primary studies on dietary patterns and longevity, (iii) we received guidance from a health information specialist with experience in conducting systematic review at the library at University of Southern Denmark, and (iv) KC and CD reviewed the search strategy. On June 4^th^, 2021, WP conducted a systematic search in the bibliographic databases “MEDLINE(R) All” and “Embase Classic + Embase”, both through the Ovid interface, covering from 1946 and 1947, respectively, until present. We build a search strategy based on the Population-Exposure-Outcome (PEO) framework consisting of three facets based on the terms “Diet”, “Longevity”, and “Elderly”. We implemented the Ovid Expert Search Filters for focused searches on elderly in both databases [39]*.* No other filters or limits were used. The search histories for Medline and Embase including search terms in full detail are presented in additional tables (see Additional Tables 1 and 2, respectively).

During the development of the search strategy, we identified four longevity projects with collected dietary data that also had an accessible homepage:

- Leiden Longevity Study at http://www.leidenlangleven.nl/en/home
- Life and Living in Advanced Age Study at https://www.fmhs.auckland.ac.nz/en/faculty/lilacs.html
- Chinese Longitudinal Health Longevity Study at https://www.icpsr.umich.edu/web/NACDA/series/487/publications and
- Japan Semi-Supercentenarian Study at http://www.keio-centenarian.com/english/research/jss

By June 4, 2021, WP searched these homepages for reports including “diet” in the title by using the search functionality in the Microsoft Edge browser.

### Eligibility criteria

We screened for prospective, retrospective, and multi-centre cohort and case control studies examining individuals ≥ 65 years at enrolment. This also included studies enrolling participants older than 65 years, e.g., ≥ 80 years of age. We employed no restrictions regarding health status or residence. Studies including adults ≥ 18 years were eligible if data on participants aged ≥ 65 years at enrolment was presented separately. We considered dietary patterns in concordance with the following definition: “The quantities, proportions, variety, or combination of different foods, drinks, and nutrients (when available) in diets, and the frequency with which they are habitually consumed” [40] regardless of the methodology used to assess the dietary pattern, e.g. by a priori defined DQI or through *a posterior* factor or cluster analysis [40, 41]. Studies reporting all-cause mortality as a single outcome or reporting this separately in studies with several outcome measures were eligible. We implemented no time-specific length of follow-up, but an age criterium. Reports were only eligible if we based on the presented methods and results could deduce that every individual in the study population or a subgroup could have reached a 100 + years of age during the follow-up period. We considered published primary studies in English, Danish, Swedish, and Norwegian without limits to year of dissemination.

### Selection process

WP performed title/abstract screening of each record imported to Covidence and subsequently assessed eligibility of the retrieved reports by full text screening. CD independently assessed three full text articles of which doubts about eligibility existed. Consensus was reached through discussion.

## Results

**Fig. 1:**
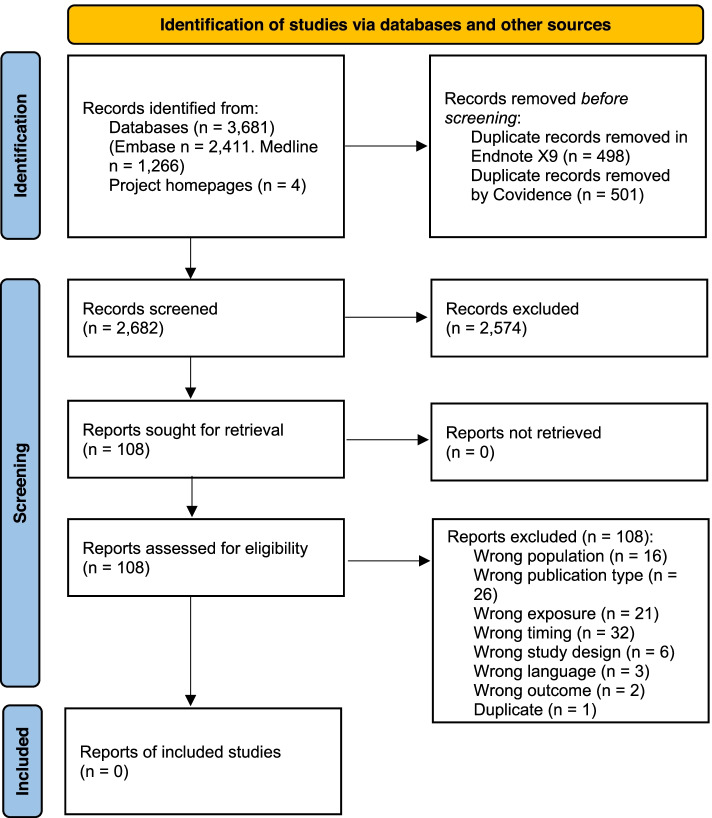
PRISMA flow diagram detailing the flow of records through the review process in the systematic review of cohort and case–control studies on dietary patterns and survival to 100 + years

The flow of records through the review process is illustrated in Fig. 1.

Our search strategy identified a total of 3,685 records, of which 999 were duplicates and therefore removed. In total 2,682 records were title/abstract-screened and 2,574 were excluded. We retrieved 108 reports, assessed eligibility by full text screening, and excluded all reports eventually. Exclusion was mainly due to studies not having long enough follow-up time to allow the cohort participants to be 100 + years, studies investigating other exposures than dietary patterns, and publications not being primary studies but for example conference abstracts and editorials. The reason for excluding each study along with citation information is presented in an additional table (See Additional table 3). Thus, the process resulted in no eligible studies for the systematic review, and consequently, since no studies were eligible, we performed no risk of bias assessment and no data synthesis.

## Discussion

This systematic review aimed to investigate the association between dietary habits in late adult life and survival to 100 + years and further to elucidate if common characteristics exist across dietary patterns associated with exceptional longevity. No studies met our inclusion criteria; hence we report an empty review, and therefore, at the current state of art, evidence remains inconclusive regarding whether dietary habits increase the probability in late life to become 100 years old.

A limited output of the systematic review process may have been expected, because studies investigating associations between diets among the oldest age groups and survival are scarce. However, it was anticipated that some of the oldest cohorts having collected dietary data, e.g., the Nurses’ Health Study [42], the Framingham Heart Study [43], and the Glostrup Population Studies [44], could have provided a first indication of whether dietary patterns continue to affect the mortality risk at high ages in the same way as observed in younger ages.

Evidence exists regarding which diets that increase our probability of surviving into old age [16, 25–27], but no longitudinal evidence exists on whether dietary habits continue to affect the chances of surviving from old to exceptional old age. It has been shown that some factors associated with higher mortality risk in adults and younger elderly lose their impact on survival among nonagenarians [45] and centenarians [46], e.g., socioeconomic status [45] and smoking [45, 46]. Therefore, it is still also debatable if the effect of healthy dietary habits on survival among adults and elderly might diminish among the oldest old populations.

Consensus exists that empty reviews are of value despite that no conclusions on the research question can be reached [35–38]. Lang et al. (2007) argues that new insights can be gained, as empty reviews “a) tell us who is undertaking the review and thus who is interested in the topic, b) highlight major research gaps, and c) indicate the state of research evidence at a particular point in time.” ([37], p. 596). It is suggested to summarize key messages from screened ineligible studies and to present observations compiled through the screening process of retrieved reports [35, 37], while being highly aware of the risk of bias, because cited studies are not selected based on inclusion criteria outlined prior to the review process [35, 36, 38].

Although this systematic review resulted in no eligible studies, we did some relevant observations during the screening of full text articles.

First, we observed that in 15 of the 108 full text screened reports follow-up had been performed at a timepoint when the entire population or a defined subgroup was ≥ 80 years of age [31, 47–60]. Thus, it seems that enough primary research has been conducted to investigate through a meta-analysis if an association exists between healthy dietary patterns and survival among the oldest old. However, this is beyond the scope of this paper. Among the 15 reports, we noted two studies which came close to being eligible for answering our research question. In one study among 1,990 participants aged ≥ 80 years from Pennsylvania, USA, the entire study population had reached ≥ 88 years of age at follow-up [58]. This represents the highest collective follow-up age identified in our systematic search. In this study, a dietary screening tool reflecting diet quality was developed and validated for the specific population. Points were assigned for both intake of specific found groups, such as whole grain breads and fruits, as well as not consuming other specific food groups, e.g., candy or chocolate, and bacon or sausages. The authors reported that having a high diet quality, defined as scoring > 75 of 105 possible points, was associated with lower mortality risk (adjusted HR = 0.76; 95% CI = 0.59–0.97; *P*-trend = 0.04) compared with having a low diet quality, i.e., scoring < 60 points. The analysis included adjustment for known potential confounders including sociodemographic covariates such as sex, age, BMI, and race, as well as variables indicating disease burden, e.g., oral health problems, and diagnosed diseases. However, the adjustment did not include income or educational attainment indicating socioeconomic status. The authors performed several secondary analyses which all suggested robustness of findings [58]. However, no subgroup analyses excluding individuals aged < 100 years at follow-up were performed. Thus, the study does not formally elucidate the impact of diet on survival to 100 + years. In another report from a study among 28,790 Chinese oldest olds, the researchers performed stratified survival analyses with individuals aged 100 + years at baseline constituting a strata [31]. A diet diversity score (DDS) was calculated for each participant with a maximum of 9 obtainable points, each point representing “often or almost daily” consumption of foods within one of 9 food groups, e.g., fish and seafood, beans, and tea. The overall finding for the entire cohort aged ≥ 80 years at baseline was a significant inverse association between the DDS and survival in the comprehensively adjusted analysis after a mean follow-up time of 3.4 years. The analysis included adjustment for sociodemographic variables such as age, sex, educational background, and living patterns, as well as lifestyle related covariates including smoking, and further considered substantial potential confounders of disease and disability, e.g., number of teeth, heart disease, and disabilities in activities of daily living. Overall, one increase in DDS was associated with a reduced mortality risk (HR = 0.91; 95% CI = 0.90–0.92; *P*-trend < 0.001). Individuals with the most diverse diets defined as a DDS ≥ 6 points had a HR of 0.56 (95% CI = 0.53–0.58) compared to individuals with the least diverse diets reflected by a score < 2 points. The effect decreased by increasing age in three strata (octogenarians, nonagenarians, and centenarians). For individuals aged ≥ 100 years at baseline one increase in DDS was associated with HR of 0.93 (95% CI = 0.92–0.94) [31]. Although including centenarians, this study does not investigate what dietary habits these individuals had while they were in their eighties or nineties, or younger. However, if increasing diet diversity is associated with better survival among octogenarians and nonagenarians, it is likely to be associated with better chances of becoming a centenarian. In other words, the nascent evidence from these two studies indicates that diets scoring high in quality or diversity continue to impact survival for individuals in their eighties, and nineties, and for centenarians.

Second, we noticed one report that investigated change in dietary habits over time among 17,959 Chinese individuals aged ≥ 65 years. Consumption of foods within 9 different food groups, e.g., fresh fruit, fish, fresh vegetables, and beans, was collected at two time points with 2–5 years between baseline and first follow-up. A diet diversity score (DDS) was calculated assigning 1 point per food group consumed 1–4 times/week and 2 points per food group consumed ≥ 5 times/week, allowing scores ranging from 0–18 points. Subsequently the relation between changes in DDS and mortality was investigated [61]. The authors reported that regardless of how the participants’ DDS changed, e.g., from low to medium or high scores, from medium to high scores, or from high to medium or low scores, the individuals maintaining high scores (13–18 points) had a lower mortality risk. The results shows that a highly diverse diet throughout several years is associated with a lower mortality risk at ages ≥ 65 years [61]. However, this study did not have a follow-up time long enough to allow the population to be 100 + years and thus does not provide information regarding if this association persists among individuals reaching 100 years of age.

Third, we identified numerous cohorts having collected data on diet among middle aged adults and/or elderly during the 1970’ies, 1980’ies, and 1990’ies (Table 1). Given that up to five decades have already passed since the establishment of these cohorts, a hundred years have passed since the entire population within some of these cohort was born. Within a decade, the same situation will occur for additional cohorts. This might allow the conduct of original studies linking collected data on diet in adulthood with mortality data and hence elucidating the association between dietary patterns in adult life and survival to 100 + years. It will, nonetheless, require very large original sample sizes to make meaningful statistical analyses due to sample attrition.

**Table 1:** Cohort studies in which dietary data has been collected. Identified through a systematic review of cohort and case control studies on dietary patterns and survival to 100 + years

Established (Year)	Study (Name, acronym)	Country(/ies)	Participants’ age at enrolment (Years)	Identified in report(s) (Author)
1948^a^	The Framingham (Heart) Study^a^ (FHS)	USA^a^	30-59^a^	^a^Dawber et al. 1951 [43] ^a^Fleming et al. 2002 [62]
1964^a^	The Glostrup Population Studies^a^	Denmark^a^	40-70^a^	^a^Hagerup et al. 1981 [44]
1971	The Gerontological and Geriatric Population Studies in Gothenburg (H7)	Sweden	70	Tognon et al. 2011 [63]
1976	Nurses’ Health Study (NHS)	USA	30–55	Lo et al. 2021 [64]
1984	Finland, Italy, the Netherlands, Elderly Studies (FINE)	Finland Italy Netherlands	65–84	Knoops et al. 2004 [60] Knoops et al. 2006 [59]
1986	The Health Professionals Follow-up Study (HPFS)	USA	40–75	Lo et al. 2021 [64]
1986	Iowa Women’s Health Study (IWHS)	USA	55–69	Inoue-Choi et al. 2013 [65] Mao et al. 2021 [53]
1988	The Survey in Europe on Nutrition and the Elderly, a Concerted Action (SENECA)	Europe	70–75	Jankovic et al. 2014 [66] Knoops et al. 2004 [60] Knoops et al. 2006 [59]
1989	The Cardiovascular Health Study (CHS)	USA	≥ 65	Diehr & Beresford 2003 [67] Greenlee et al. 2014 [52]
1989	Nurses’ Health Study II (NHSII)	USA	25–42	Lo et al. 2021 [64]
1990^a^	European Prospective Investigation Into Cancer and Nutrition – Elderly (EPIC-Elderly)	UK Germany Netherlands France Spain Italy Greece Sweden Denmark ^a^	35-74^a^	Jankovic et al. 2014 [66] ^a^Riboli & Kaaks 1997 [68]
1990^a^	The Rotterdam Elderly Study	Netherlands	≥ 55^a^	Jankovic et al. 2014 [66] ^a^Hofman et al. 1991 [69]
1992	Blue Mountains Eye Study (BMES)	Australia	≥ 49	Russel et al. 2013 [70]
1992	The Italian Longitudinal Study on Ageing (ILSA)	Italy	65–84	Limongi et al. 2017 [71]
1994	The Geisinger Rural Aging Study (GRAS)	USA	≥ 65	Liu et al. 2019 [58]
1994	National Diet and Nutrition Survey	UK	≥ 65	Hamer et al. 2010 [72]
1995	The National Institutes of Health-AARP Diet and Health (NIH-AARP)	USA	50–71	Jankovic et al. 2014 [66] Reedy et al. 2014 [73]
1996	The New Integrated Suburban Seniority Investigation Project (NISSIN)	Japan	64–65	Sasakabe et al. 2021 [55]
1998	The Chinese Longitudinal Healthy Longevity Survey (CLHLS)	China	≥ 65	Shi et al. 2015 [74] Cao et al. 2019 [57] Lv et al. 2020 [31] Liu et al. 2021 [61]
2009^a^	The Mugello Study^a^	Italy^a^	90-99^a^	^a^Molino-Lova et al. 2013 [75]

^a^Information not available in screened reports and therefore retrieved from other sources

This systematic review has limitations. First, only one author screened titles and abstracts which may have resulted in missed relevant studies [34]. However, this seems unlikely as 108 papers were retrieved and full text-screened to assure that no studies had relevant subgroup or supplementary analyses concordant with our eligibility criteria. Second, we only looked for published studies and some unpublished information might be found in the above-mentioned cohorts already. Third, we chose to search for studies with cohort and case–control designs, because we expected to find at least a few eligible studies. With the review process coming up empty, we could have included lower quality evidence such as cross-sectional studies. However, as we aimed to elucidate if dietary patterns affect the probability of surviving to 100 years, cross-sectional studies will not suffice. Fourth, the largest exclusion of studies was caused by the inclusion criteria of long enough follow-up time to allow participants to survive to 100 + years as a definition of exceptional longevity. However, exceptional longevity could be defined otherwise [9]. Another option would have been to choose a top x% oldest of a specific sex- and birth cohort where the age defined as exceptional longevity was set by the implemented percentage, e.g., the top 1% oldest American males born in 1900 [9]. Implementing a definition of exceptional survival as a top x% of survivors, eligible primary studies would have to have results at follow-up ages corresponding to the chosen percentage and of the chosen sex to be comparable. The follow-up age would then vary across cohorts because different birth cohorts in different areas of the world have been impacted by different secular trends [9, 76]. However, this method to define exceptional survival and follow-up ages has not been implemented in any of the excluded cohort studies. A third opportunity for defining exceptional longevity could be to lower the age threshold from 100 to, e.g., 90 years. This would potentially increase the probability of finding eligible studies, as a shorter follow-up period would be required. However, while there are numerous centenarian studies worldwide that search for determinants of longevity, there are few studies using 90 years as eligibility criteria. Furthermore, centenarians are a much more selected group than 90-year-olds. In the Danish 1905 cohort the probability of surviving from birth to 92 years (1 in 20) is about the same as surviving from age 93 to 100 years (again 1 in 20) [77].

## Conclusion

This systematic review resulted in no eligible studies that met our a priori chosen inclusion criteria and hence we have reported an empty review. Consequently, no direct evidence currently exists on the association between dietary habits in late adult life and survival to 100 + years. We have identified several cohorts worldwide where data on diet has been collected during the participants’ adult lives, and where a century has passed since the participants were born. Thus, cohort studies combining already collected dietary data with updated mortality data could be conducted within these cohorts. Attention should be directed at the sizes of the original cohorts to ensure sufficient power after sample attrition.

## Supplementary Information

**Additional file 1.** Documentation of conformity to the PRISMA 2020 in reporting the systematic review of cohort and case-control studies on dietary patterns and survival to 100+ years.

**Additional file 2: Additional Table 1.** Search history of Ovid MEDLINE(R) All 1946 to 4 June 2021 in the systematic review of cohort and case-c`ontrol studies on dietary patterns and survival to 100+ years.

**Additional file 3: Additional Table 2.** Search history of Ovid Embase Classic+Embase 1947 to 4 June 2021 in the systematic review of cohort and case-control studies on dietary patterns and survival to 100+ years.

**Additional file 4: Additional Table 3.** Distribution of 108 records assessed by full-text review across unfulfilled eligibility criteria in the systematic review of cohort and case-control studies on dietary patterns and survival to 100+ years.

## Data Availability

Data sharing is not applicable to this article as no datasets were generated or analyzed during the current study.
