# Supplementary material for: Dietary patterns and survival to 100 + years: an empty systematic review of cohort and case–control studies
Source: Arch Public Health. 2022 Jun 29;80:161. doi: 10.1186/s13690-022-00914-2 (PMC9241213; doi:10.1186/s13690-022-00914-2)
Supplement: Supplementary file 2 — Additional file 2: Additional Table 1. Search history of Ovid MEDLINE(R) All 1946 to 4 June 2021 in the systematic review of cohort and case-c`ontrol studies on dietary patterns and survival to 100+ years. [file 13690_2022_914_MOESM2_ESM.docx]

# Additional file 2

**Additional table 1.** Search history of Ovid MEDLINE(R) All 1946 to 4 June 2021 in the systematic review of cohort and case-control studies on dietary patterns and survival to 100+ years.

| # | **Searches** | **Results** |
| --- | --- | --- |
| 1 | exp diet/ or diet*.mp. | 834599 |
| 2 | exp longevity/ or exp mortality/ or longevity.mp. or overall survival.mp. or mortality.mp. | 1481363 |
| 3 | exp *aged/ or exp *geriatrics/ or exp *geriatric nursing/ or (centarian* or centenarian* or elder* or eldest or frail* or geriatri* or nonagenarian* or octagenarian* or octogenarian* or old age* or older adult* or older age* or older female* or older male* or older man or older men or older patient* or older people or older person* or older population or older subject* or older woman or older women or oldest old* or senior* or senium or septuagenarian* or supercentenarian* or very old*).ti,kf. | 295336 |
| 4 | 1 and 2 and 3 | 1266 |

*Note: * truncation*
