# Supplementary material for: Dietary patterns and survival to 100 + years: an empty systematic review of cohort and case–control studies
Source: Arch Public Health. 2022 Jun 29;80:161. doi: 10.1186/s13690-022-00914-2 (PMC9241213; doi:10.1186/s13690-022-00914-2)
Supplement: Supplementary file 4 — Additional file 4: Additional Table 3. Distribution of 108 records assessed by full-text review across unfulfilled eligibility criteria in the systematic review of cohort and case-control studies on dietary patterns and survival to 100+ years. [file 13690_2022_914_MOESM4_ESM.docx]

# Additional file 4

**Additional table 3.** Distribution of 108 records assessed by full-text review across unfulfilled eligibility criteria in the systematic review of cohort and case-control studies on dietary patterns and survival to 100+ years.

| **Eligibility criteria not fulfilled** | **Number and reference** |
| --- | --- |
| Population | N=16 (1-16) |
| Publication type | N=26 (17-42) |
| Exposure | N=21 (43-64) |
| Timing | N=32 (65-96) |
| Study design | N=6 (97-102) |
| Language | N=3 (103-105) |
| Outcome | N=2 (106, 107) |
| Manually identified duplicate | N=1 (72) |

# Reference list

1. Bamia C, Kasapa C, Orfanou A, Travezea C, Trichopoulou A, Trichopoulos D, et al. Dietary patterns and survival of older Europeans: The EPIC-Elderly Study (European Prospective Investigation into Cancer and Nutrition). Public health nutrition. 2007;10(6):590-8.

2. Carballo-Casla A, Ortola R, Garcia-Esquinas E, Oliveira A, Lopes C, Sotos-Prieto M, et al. The Southern European Atlantic Diet and all-cause mortality in older adults. BMC Medicine. 2021;19(1):36.

3. Frisoni GB, Franzoni S, Rozzini R, Ferrucci L, Boffelli S, Trabucchi M. Food intake and mortality in the frail elderly. J Gerontol A Biol Sci Med Sci. 1995;50(4):M203-10.

4. Inoue-Choi M, Robien K, Lazovich D. Adherence to the WCRF/AICR guidelines for cancer prevention is associated with lower mortality among older female cancer survivors. Cancer epidemiology, biomarkers & prevention : a publication of the American Association for Cancer Research, cosponsored by the American Society of Preventive Oncology. 2013;22(5):792-802.

5. Jankovic N, Geelen A, Streppel MT, de Groot LCPGM, Orfanos P, van den Hooven EH, et al. Adherence to a healthy diet according to the World Health Organization guidelines and all-cause mortality in elderly adults from Europe and the United States. Am J Epidemiol. 2014;180(10):978-88.

6. Jayanama K, Theou O, Blodgett JM, Cahill L, Rockwood K. Frailty, nutrition-related parameters, and mortality across the adult age spectrum. BMC Medicine. 2018;16(1):188.

7. Jayanama K, Theou O, Godin J, Cahill L, Shivappa N, Hebert JR, et al. Relationship between diet quality scores and the risk of frailty and mortality in adults across a wide age spectrum. BMC Medicine. 2021;19(1):64.

8. Mao Z, Prizment AE, Lazovich D, Bostick RM. Associations of dietary and lifestyle oxidative balance scores with mortality risk among older women: the Iowa Women's Health Study. European journal of nutrition. 2021.

9. Reedy J, Krebs-Smith SM, Subar AF, Park Y, Miller PE, Liese AD, et al. Higher diet quality is associated with decreased risk of all-cause, cardiovascular disease, and cancer mortality among older adults. Journal of Nutrition. 2014;144(6):881-9.

10. Russell J, Flood V, Rochtchina E, Gopinath B, Mitchell P, Allman-Farinelli M, et al. Adherence to dietary guidelines and 15-year risk of all-cause mortality. British Journal of Nutrition. 2013;109(3):547-55.

11. Sasakabe T, Wakai K, Ukawa S, Ando M, Kawamura T, Okabayashi S, et al. Food group intakes and all-cause mortality among a young older Japanese population of the same age: the New Integrated Suburban Seniority Investigation Project. Nagoya journal of medical science. 2021;83(1):169-82.

12. Schooling CM, Ho SY, Leung GM, Thomas GN, McGhee SM, Lam TH, et al. Diet synergies and mortality - A population-based case-control study of 32 462 Hong Kong Chinese older adults. International journal of epidemiology. 2006;35(2):418-26.

13. Shimizu K, Takeda S, Noji H, Hirose N, Ebihara Y, Arai Y, et al. Dietary patterns and further survival in Japanese centenarians. Journal of Nutritional Science and Vitaminology. 2003;49(2):133-8.

14. Trichopoulou A, Orfanos P, Norat T, Bueno-de-Mesquita B, Ocke MC, Peeters PHM, et al. Modified Mediterranean diet and survival: EPIC-elderly prospective cohort study. BMJ (Clinical research ed). 2005;330(7498):991.

15. Zhao W, Ukawa S, Tamakoshi A, Okada E, Wakai K, Kawamura T, et al. The associations of dietary patterns with all-cause mortality and other lifestyle factors in the elderly: An age-specific prospective cohort study. Clinical Nutrition. 2019;38(1):288-96.

16. Lo C-H, Khalili H, Song M, Lochhead P, Burke KE, Richter JM, et al. Healthy Lifestyle Is Associated With Reduced Mortality in Patients With Inflammatory Bowel Diseases. Clinical Gastroenterology and Hepatology. 2021;19(1):87-95.e4.

17. Anonymous. Mediterranean diet leads to longer life for elderly. Pharmaceutical Journal. 2004;273(7319):455.

18. Anonymous. Health after 100: secrets of the centenarians. The Johns Hopkins medical letter health after 50. 2001;13(9):6-7.

19. Caruso C, Passarino G, Puca A, Scapagnini G. "Positive biology": The centenarian lesson. Immunity and Ageing. 2012;9(1):5.

20. Chrysohoou C, Skoumas J, Lazaros G, Katte K, Mazaris S, Venieri E, et al. Long-term nutritional habits and four-year (2009-2013) all cause and cardiovascular disease mortality in older adults: The Ikaria study. European Heart Journal. 2015;36(SUPPL. 1):114.

21. Cowley G. How to live to 100. Newsweek. 1997;129(26):56-67.

22. DeSilvey DL. Diet, lifestyle, mortality, and memory in the elderly. The American journal of geriatric cardiology. 2005;14(1):41.

23. Forette B. Centenarians' food behavior. Cahiers de Nutrition et de Dietetique. 1996;31(2):102-8.

24. Foskolou A, Matalas A, Polychronopoulos E, Sidossis L, Panagiotakos DB, Tyrovolas S, et al. Successful ageing-continental vs insular Mediterranean areas and Western vs Eastern Greek Mediterranean islands-of older adults: The multinational MEDIS study. European Heart Journal. 2017;38(Supplement 1):721.

25. Fraser GE, Shavlik DJ. Some lifestyle and dietary habits were associated with all-cause and coronary heart disease mortality in elderly persons. Evidence-Based Medicine. 1998;3(4):125.

26. Gearon CJ. A ripe old age. The right foods, moderate exercise, and regular medical checkups can add years to your life. US news & world report. 2005;138(22):46-7.

27. Hamer M, Bates CJ, Mishra GD. Multiple health behaviors and mortality risk in older adults. J Am Geriatr Soc. 2011;59(2):370-2.

28. Horng MS, Knoops KT, de Groot LC, Kromhout D. Healthy lifestyle and mediterranean diet decreases mortality in the elderly. Journal of Clinical Outcomes Management. 2004;11(11):688-9.

29. Inoue-Choi M, Lazovich D, Robien K. Adherence to the WCRF/AICR recommendations for cancer prevention is associated with all-cause and cancer mortality among elderly female cancer survivors. Cancer Prevention Research. 2012;5(11 SUPPL. 1).

30. Jankovic N, Geelen A, Kampman E, De Groot CP, Feskens EJ, Pikhart H, et al. Association between a healthy diet according to who guidelines and all-cause mortality in european and american elderly, the chances project. Annals of Nutrition and Metabolism. 2013;63(SUPPL. 1):234.

31. Knoops KTB, Roth Z, Brown G, de Groot LCPGM, Kromhout D, Perrin AE, et al. Mediterranean diet, lifestyle factors, and 10-year mortality in elderly European men and women: The HALE project. Evidence-Based Ophthalmology. 2005;6(1):48-9.

32. Martinez-Gomez D, Guallar-Castillon P, Leon-Munoz LM, Lopez-Garcia E, Rodriguez-Artalejo F. Combined effect of traditional and nontraditional health behaviors on mortality in Spanish older adults. Annals of Nutrition and Metabolism. 2013;63(SUPPL. 1):1066.

33. Perez-Matos MC, Mair WB. Predicting longevity responses to dietary restriction: A stepping stone toward precision geroscience. PLoS Genetics. 2020;16(7):e1008833.

34. Perls T. The different paths to age one hundred. Annals of the New York Academy of Sciences. 2005;1055:13-25.

35. Rathod AD, Bharadwaj AS, Afonso L, Kizilbash M, Badheka AO. Healthy eating index and mortality in a nationally representative elderly cohort. Archives of Internal Medicine. 2012;172(3):275-7.

36. Steen B. A 'healthy' lifestyle in old age and its relation to health and disease. Age and Ageing. 2003;32(4):365-6.

37. Verheijden MW, Van Staveren WA, De Henauw S, Schroll M, De Groot LCPMG. Lifestyle, nutritional status, health, and mortality in elderly people across Europe: A review of the longitudinal results of the SENECA study. Journals of Gerontology - Series A Biological Sciences and Medical Sciences. 2004;59(12):1277-84.

38. Waddington GS. Daily steps and diet, but not sleep, are related to mortality in older Australians. Journal of Science and Medicine in Sport. 2020;23(3):207.

39. Wu IC, Hahn L-C, Lin X-Z. Longevity and successful aging. Journal of Internal Medicine of Taiwan. 2008;19(5):394-400.

40. Xiong S, Li Y, Yan L. Lifestyle factors substantially contributed to urban-rural disparity in all-cause mortality among the oldest-old in China. Circulation. 2019;139(Supplement 1).

41. Zanni GR, Wick JY. Centenarians: 100 Years and beyond. Consultant Pharmacist. 2004;19(10):864-70.

42. Bonaccio M, Di Castelnuovo A, Costanzo S, Persichillo M, Donati MB, De Gaetano G, et al. Higher adherence to the traditional Mediterranean diet is associated with lower cardiovascular risk and all-cause mortality in the elderly: Prospective findings from the Moli-sani study. European Journal of Preventive Cardiology. 2017;24(1 Supplement 1):S8.

43. Ando T, Nakazato K, Kiko T, Shimizu T, Yoshihisa A, Yamaki T, et al. Impact of geriatric nutrition risk index on long-term prognosis in patients with acute myocardial infarction. Circulation. 2019;140(Supplement 1).

44. Bates CJ, Hamer M, Mishra GD. A study of relationships between bone-related vitamins and minerals, related risk markers, and subsequent mortality in older British people: The National Diet and Nutrition Survey of People Aged 65 Years and over. Osteoporosis International. 2012;23(2):457-66.

45. Berr C, Hininger-Favier I, Roussel A-M, Gourlet V, Arnaud J, Akbaraly NT. Selenium and mortality in the elderly: Results from the EVA study. Clinical Chemistry. 2005;51(11):2117-23.

46. Jayanama K, Theou O, Godin J, Cahill L, Rockwood K. Association of fatty acid consumption with frailty and mortality among middle-aged and older adults. Nutrition (Burbank, Los Angeles County, Calif). 2020;70:110610.

47. Kaluza J, Dolowa J, Roszkowski W, Brzozowska A. Survival and habitual nutrient intake among elderly men. Roczniki Panstwowego Zakladu Higieny. 2005;56(4):361-70.

48. Kwok T, Zhu ZLY, Chan RSM, Woo J, Yeung SSY. Prospective Analysis of Fruit and Vegetable Variety on Health Outcomes in Community-Dwelling Chinese Older Adults. Journal of Nutrition, Health and Aging. 2021.

49. Leung J, Woo J, Chan R. High Protein Intake Is Associated with Lower Risk of All-Cause Mortality in Community-Dwelling Chinese Older Men and Women. Journal of Nutrition, Health and Aging. 2019;23(10):987-96.

50. Liu ZM, Tse LA, Chan D, Wong C, Wong SYS. Dietary sugar intake was associated with increased body fatness but decreased cardiovascular mortality in Chinese elderly: An 11-year prospective study of Mr and Ms OS of Hong Kong. International Journal of Obesity. 2018;42(4):808-16.

51. Liu Z-M, Tse SLA, Chen B, Chan D, Wong C, Woo J, et al. Dietary sugar intake does not pose any risk of bone loss and non-traumatic fracture and is associated with a decrease in all-cause mortality among Chinese elderly: Finding from an 11-year longitudinal study of Mr. and Ms. OS Hong Kong. Bone. 2018;116:154-61.

52. Magni E, Bianchetti A, Rozzini R, Trabucchi M. Influence of nutritional intake on 6-year mortality in an Italian elderly population. Journal of nutrition for the elderly. 1994;13(4):25-34.

53. Meyer KA, Pereira MA, Kushi LH, Jacobs Jr DR. Fiber from whole grains, but not refined grains, is inversely associated with all-cause mortality in older women: The Iowa Women's Health Study. Journal of the American College of Nutrition. 2000;19(3 SUPPL.):326S-30S.

54. Meyer KA, Kushi LH, Folsom AR, Jacobs Jr DR. Is whole grain intake associated with reduced total and cause-specific death rates in older women? The Iowa women's health study. Am J Public Health. 1999;89(3):322-9.

55. Milder IEJ, Feskens EJM, Arts ICW, Bueno-de-Mesquita HB, Hollman PCH, Kromhout D. Intakes of 4 dietary lignans and cause-specific and all-cause mortality in the Zutphen Elderly Study. The American journal of clinical nutrition. 2006;84(2):400-5.

56. Sahyoun NR, Jacques PF, Russell RM. Carotenoids, vitamins C and E, and mortality in an elderly population. Am J Epidemiol. 1996;144(5):501-11.

57. Fraser GE, Shavlik DJ. Risk Factors for All-Cause and Coronary Heart Disease Mortality in the Oldest-Old: The Adventist Health Study. Archives of Internal Medicine. 1997;157(19):2249-58.

58. Tani Y, Suzuki N, Fujiwara T, Hanazato M, Kondo N, Miyaguni Y, et al. Neighborhood food environment and mortality among older Japanese adults: results from the JAGES cohort study. The international journal of behavioral nutrition and physical activity. 2018;15(1):101.

59. Toffanello ED, Inelmen EM, Sergi G, Coin A, Miotto F, Enzi G, et al. Ten-year trends in dietary intake, health status and mortality rates in free-living elderly people. Journal of Nutrition, Health and Aging. 2010;14(4):259-64.

60. Tognon G, Petrolo M, Sundh V, Lissner L, Rothenberg E. Dairy product intake and mortality in a cohort of 70-year-old Swedes: a contribution to the Nordic diet discussion. European journal of nutrition. 2018;57(8):2869-76.

61. Wang XJ, Jiang CQ, Zhang WS, Zhu F, Jin YL, Woo J, et al. Milk consumption and risk of mortality from all-cause, cardiovascular disease and cancer in older people. Clinical nutrition (Edinburgh, Scotland). 2020;39(11):3442-51.

62. Shi Z, Martin S, Avery JC, Taylor AW, Zhang T, Byles J. Food habits, lifestyle factors and mortality among oldest old Chinese: The Chinese longitudinal healthy longevity survey (CLHLS). Nutrients. 2015;7(9):7562-79.

63. Liu D, Zhang X-R, Li Z-H, Zhang Y-J, Wang Z-H, Shen D, et al. Association of dietary diversity changes and mortality among older people: A prospective cohort study. Clinical Nutrition. 2021;40(5):2620-9.

64. Ding D, Rogers K, van der Ploeg H, Stamatakis E, Bauman AE. Traditional and Emerging Lifestyle Risk Behaviors and All-Cause Mortality in Middle-Aged and Older Adults: Evidence from a Large Population-Based Australian Cohort. PLoS medicine. 2015;12(12):e1001917.

65. Bonaccio M, Di Castelnuovo A, Costanzo S, Gialluisi A, Persichillo M, Cerletti C, et al. Mediterranean diet and mortality in the elderly: a prospective cohort study and a meta-analysis. The British journal of nutrition. 2018;120(8):841-54.

66. Anderson AL, Harris TB, Tylavsky FA, Perry SE, Houston DK, Hue TF, et al. Dietary Patterns and Survival of Older Adults. Journal of the American Dietetic Association. 2011;111(1):84-91.

67. Bonaccio M, Di Castelnuovo A, Costanzo S, De Curtis A, Persichillo M, Cerletti C, et al. Impact of combined healthy lifestyle factors on survival in an adult general population and in high-risk groups: prospective results from the Moli-sani Study. Journal of internal medicine. 2019;286(2):207-20.

68. Brown JC, Harhay MO, Harhay MN. Physical activity, diet quality, and mortality among sarcopenic older adults. Aging Clinical and Experimental Research. 2017;29(2):257-63.

69. Brown JC, Harhay MO, Harhay MN. Physical Activity, Diet Quality, and Mortality among Community-Dwelling Prefrail and Frail Older Adults. Journal of nutrition in gerontology and geriatrics. 2016;35(4):253-66.

70. Cao Z, Cheng Y, Li S, Sun L, Wang Y, Wang R, et al. Adherence to a healthy lifestyle counteracts the negative effects of risk factors on all-cause mortality in the oldest-old. Aging (Albany NY). 2019;11(18):7605-19.

71. Chen RC-Y, Chang Y-H, Lee M-S, Wahlqvist ML. Dietary quality may enhance survival related to cognitive impairment in Taiwanese elderly. Food and Nutrition Research. 2011;55:7387.

72. Chrysohoou C, Pitsavos C, Lazaros G, Skoumas J, Tousoulis D, Stefanadis C, et al. Determinants of All-Cause Mortality and Incidence of Cardiovascular Disease (2009 to 2013) in Older Adults: The Ikaria Study of the Blue Zones. Angiology. 2016;67(6):541-8.

73. Diehr P, Beresford SAA. The relation of dietary patterns to future survival, health, and cardiovascular events in older adults. Journal of clinical epidemiology. 2003;56(12):1224-35.

74. Fortes C, Forastiere F, Farchi S, Rapiti E, Pastori G, Perucci CA. Diet and overall survival in a cohort of very elderly people. Epidemiology (Cambridge, Mass). 2000;11(4):440-5.

75. Greenlee H, Lovasi GS, Richardson J, Fried LP, Strizich G, Kaplan RC, et al. Concordance with Prevention Guidelines and Subsequent Cancer, Cardiovascular Disease, and Mortality: A Longitudinal Study of Older Adults. Am J Epidemiol. 2017;186(10):1168-79.

76. Hamer M, McNaughton SA, Bates CJ, Mishra GD. Dietary patterns, assessed from a weighed food record, and survival among elderly participants from the United Kingdom. European journal of clinical nutrition. 2010;64(8):853-61.

77. Hays JC, Keller HH, Ostbye T. The effects of nutrition-related factors on four-year mortality among a biracial sample of community-dwelling elders in the North Carolina piedmont. Journal of nutrition for the elderly. 2005;25(2):41-67.

78. Knoops KTB, de Groot LC, van Staveren WA, Fidanza F, Alberti-Fidanza A, Kromhout D. Comparison of three different dietary scores in relation to 10-year mortality in elderly European subjects: The HALE project. European journal of clinical nutrition. 2006;60(6):746-55.

79. Knoops KTB, de Groot LCPGM, Kromhout D, Perrin A-E, Moreiras-Varela O, Menotti A, et al. Mediterranean diet, lifestyle factors, and 10-year mortality in elderly European men and women: the HALE project. JAMA. 2004;292(12):1433-9.

80. Kouris-Blazos A, Wahlqvist ML, Lukito W, Gnardellis C, Trichopoulou A, Trichopoulos D. Are the advantages of the mediterranean diet transferable to other populations? A cohort study in Melbourne, Australia. British Journal of Nutrition. 1999;82(1):57-61.

81. Kumagai S, Shibata H, Watanabe S, Suzuki T, Haga H. Effect of food intake pattern on all-cause mortality in the community elderly: a 7-year longitudinal study. The journal of nutrition, health & aging. 1999;3(1):29-33.

82. Lampropoulos CE, Konsta M, Dradaki V, Roumpou A, Dri I, Papaioannou I. Effects of Mediterranean diet on hospital length of stay, medical expenses, and mortality in elderly, hospitalized patients: A 2-year observational study. Nutrition. 2020;79-80:110868.

83. Limongi F, Noale M, Crepaldi G, Maggi S, Gesmundo A. Adherence to the Mediterranean Diet and all-cause mortality risk in an elderly Italian population: Data from the ILSA study. Journal of Nutrition, Health and Aging. 2017;21(5):505-13.

84. Liu Y-H, Gao X, Mitchell DC, Wood GC, Still CD, Jensen GL. Diet Quality Is Associated With Mortality in Adults Aged 80 Years and Older: A Prospective Study. J Am Geriatr Soc. 2019;67(10):2180-5.

85. Lundin H, Strender LE, Mollasaraie HA, Salminen H, Saaf M. Mini nutritional assessment and 10-year mortality in free-living elderly women: A prospective cohort study with 10-year follow-up. European journal of clinical nutrition. 2012;66(9):1050-3.

86. Lv Y, Zhou J, Shi W, Shi X, Kraus VB, Gao X, et al. Higher dietary diversity scores and protein-rich food consumption were associated with lower risk of all-cause mortality in the oldest old. Clinical Nutrition. 2020;39(7):2246-54.

87. Martinez-Gomez D, Guallar-Castillon P, Leon-Munoz LM, Lopez-Garcia E, Rodriguez-Artalejo F. Combined impact of traditional and non-traditional health behaviors on mortality: a national prospective cohort study in Spanish older adults. BMC medicine. 2013;11:47.

88. Mitchell DC, Hartman TJ, Coffman DL, Craig Wood G, Still C, Hsiao PY, et al. Dietary patterns and relationship to obesity-related health outcomes and mortality in adults 75 years of age or greater. Journal of Nutrition, Health and Aging. 2013;17(6):566-72.

89. Ramage-Morin PL, Gilmour H, Rotermann M. Nutritional risk, hospitalization and mortality among community-dwelling Canadians aged 65 or older. Health reports. 2017;28(9):17-27.

90. Soderstrom L, Rosenblad A. Association between separate items of the Mini Nutritional Assessment instrument and mortality among older adults: A prospective cohort study introducing a trimmed MNA version. Clinical Nutrition. 2020;39(7):2255-64.

91. Stanaway FF, Ribeiro RV, Khalatbari-Soltani S, Cvejic E, Blyth FM, Naganathan V, et al. Diet quality in an ethnically diverse population of older men in Australia. European journal of clinical nutrition. 2021.

92. Tognon G, Rothenberg E, Eiben G, Sundh V, Winkvist A, Lissner L. Does the Mediterranean diet predict longevity in the elderly? A Swedish perspective. Age (Dordrecht, Netherlands). 2011;33(3):439-50.

93. Trichopoulou A, Kouris-Blazos A, Wahlqvist ML, Gnardellis C, Lagiou P, Polychronopoulos E, et al. Diet and overall survival in elderly people. BMJ (Clinical research ed). 1995;311(7018):1457-60.

94. Wahlqvist ML, Kouris-Blazos A, Steen B, Lukito W, Horie Y, Horie K, et al. Legumes: The most important dietary predictor of survival in older people of different ethnicities. Asia Pacific journal of clinical nutrition. 2004;13(2):217-20.

95. Wong M, Woo J, Chan R, Leung J. Relative contributions of geographic, socioeconomic, and lifestyle factors to quality of life, frailty, and mortality in elderly. PLoS One. 2010;5(1):e8775.

96. Woo J, Ho SC, Yu ALM. Lifestyle factors and health outcomes in elderly Hong Kong chinese aged 70 years and over. Gerontology. 2002;48(4):234-40.

97. Cardenas-Fuentes G, Subirana I, Schroder H, Fito M, Ruiz-Canela M, Martinez-Gonzalez MA, et al. Multiple approaches to associations of physical activity and adherence to the Mediterranean diet with all-cause mortality in older adults: the PREvencion con DIeta MEDiterranea study. European journal of nutrition. 2019;58(4):1569-78.

98. Foscolou A, Magriplis E, Soulis G, Bountziouka V, Gotsis E, Metallinos G, et al. Lifestyle determinants of healthy ageing in a Mediterranean population: The multinational MEDIS study. Experimental gerontology. 2018;110:35-41.

99. Pes GM, Errigo A, Concu D, Tolu F, Chambre D, Poulain M. Genetic and non-genetic factors associated with population longevity in Sardinia. European Geriatric Medicine. 2014;5(SUPPL. 1):S148.

100. Sourander LB, Ruikka I, Kasanen A. A health survey on the aged with a 5-year follow-up. Acta socio-medica Scandinavica Supplement. 1970;3:1-41.

101. Trichopoulou A, Kouris-Blazos A, Vassilakou T, Gnardellis C, Polychronopoulos E, Venizelos M, et al. Diet and survival of elderly greeks: A link to the past. American Journal of Clinical Nutrition. 1995;61(6 SUPPL.):1346S-50S.

102. Xiao Z, Xu Q, Yuan Y. Solving the mystery of the status and longevity of centenarians in Bama. Chinese journal of population science. 1996;8(4):385-94.

103. Frackiewicz J, Kaluza J, Roszkowski W, Brzozowska A. Influence of chosen lifestyle and diet factors on mortality among elderly people living in Warsaw and surrounding villages. Przeglad epidemiologiczny. 2009;63(3):431-5.

104. Frackiewicz J, Roszkowski W, Brzozowska A, Kaluza J. Diet quality and mortality in elderly people living in Warsaw Region. Przeglad epidemiologiczny. 2010;64(1):119-25.

105. Bourdel-Marchasson I, Traissac T. Indications and possible consequences of diets in the oldest old. Nutrition Clinique et Metabolisme. 2004;18(4):224-30.

106. Jankovic N. WHO guidelines for a healthy diet and mortality from cardiovascular disease in European and American elderly: The chances project. European Journal of Epidemiology. 2015;30(8):729.

107. Lo Buglio A, Bellanti F, Capurso C, Paglia A, Vendemiale G. Adherence to Mediterranean Diet, Malnutrition, Length of Stay and Mortality in Elderly Patients Hospitalized in Internal Medicine Wards. Nutrients. 2019;11(4).
